# Allelopathic effects of tree peony extracts on bok choy, spinach and tatsoi

**DOI:** 10.1038/s41598-025-11422-y

**Published:** 2025-12-09

**Authors:** Yingzi Guo, Songlin He, Shulin Zhang, Laikun Shi, Wenqing Jia

**Affiliations:** 1 College of Horticulture and Landscape Architecture, Henan Institute of Science and Technology, Xinxiang, 453003 China; 2 College of Landscape Architecture and Art, Henan Agricultural University, Zhengzhou, 450002 China; 3 College of geography and geomatics, Xuchang University, Xuchang, 461000 China

**Keywords:** Ecology, Physiology, Plant sciences

## Abstract

Although tree peony (*Paeonia ostii*) is widely cultivated in China, it has not been fully utilized as an arable resource. To investigate the suitability of tree peony in intercropping systems, we examined the allelopathic effects of aqueous extracts from the leaves and roots of *P. ostii* ‘Fengdan’ on the seed germination, growth, and physiological responses of tatsoi, spinach, and bok choy. Among these vegetables, tatsoi exhibited the best seed germination and seedling growth. Lower concentrations of aqueous extracts of ‘Fengdan’ roots (5–10 g L^−1^ for tatsoi and 5 g L^−1^ for spinach and bok choy) promoted seed germination, biomass, chlorophyll content, root activity, photosynthetic rate, intercellular CO_2_ concentration, stomatal conductance, and antioxidant enzyme activity. However, high concentrations of the leaf extract (40 g L^−1^ for tatsoi and 20–40 g L^−1^ for spinach and bok choy) significantly increased levels of malondialdehyde and reduced seed germination, dry weight, chlorophyll content, root activity, and antioxidant enzyme activity. These deleterious effects increased with higher extract concentrations. The allelopathic effect of the leaf extract was greater than that of the root extract. The order of the allelopathic effect of tree peonies on the three vegetables was bok choy, spinach, and tatsoi. Therefore, tatsoi is the most suitable vegetable for interplanting with tree peonies.

## Introduction

Allelopathy is an important ecological phenomenon in which biochemicals released by one plant can affect the growth of other plants^1,2^. Vegetable agroforestry farming systems (VAFS) are a model of crop management that aims to increase food production and more efficiently utilize arable land. VAFS involves the cultivation of woody plants and vegetable crops in specified temporal or spatial arrangements^3–5^. The allelopathic compatibility between vegetable crops and woody plants may be key to the success of a vegetable agroforestry system^4,6^. Woody plants release a number of biochemicals into the soil that inhibit the germination and development of understory crops^7,8^. There have been a number of studies in which researchers have investigated tree-crop interactions in cereal based systems^7–9^; however, there is as yet little information about the interactions between trees and vegetables, which is essential for planning VAFS. It has been reported that most of the tree species used in agroforestry have negative impacts on the growth of food crops^7,8,10^. For example, leaf extracts from four woody plants, including *Acacia auriculiformi* and *Tectona grandis,* were reported to inhibit the germination rate of tomato, brinjal, and chilli; inhibition of radical and plumule growth was more pronounced in these systems^11^. The authors hypothesized that certain compounds from these tree species may negatively affect the physiology of understory crops. Lawrence et al.^12^ reported that aqueous extracts of leaves and stems of *Ailanthus altissima* negatively affect seed germination and seedling growth of eight North American native plants. Bauer et al.^13^ found that leaf extracts from *Lonicera maackii* inhibited seed germination in several herbaceous plants: *Bredia hirsuta, Alliaria petiolata*, and *Impatiens capensis*. Furthermore, *Phyllostachys edulis* was found to contain high levels of allelopathic compounds that inhibit the growth of lower weeds^14^.

The level of malondialdehyde (MDA) in a plant reflects the balance of oxidative stress. Since allelochemicals affect antioxidant enzymes and MDA levels, MDA measurements are used to indicate oxidative damage to biomaterials^15–17^. The process by which superoxide dismutase (SOD) dismutates O_2_^−^ to H_2_O_2_ by integration with metal ions is considered the first line of the antioxidant defense system^18,19^. Peroxidase (POD) and catalase (CAT) mainly remove H_2_O_2_ and maintain the intracellular concentration of H_2_O_2_ within a controllable range^20,21^. Researchers have shown that plants generate large amounts of ROS under stress from allelopathic biochemicals released by other plants in intercropping systems^22–24^. The increase in MDA levels during germination of Jatropha seeds is associated with increased POD, SOD, and CAT activity^25^. The effect of these allelopathic biochemicals on the growth of other plants is unclear. Therefore, further study in this field is needed to promote the growth of vegetables or crops in VAFS where companion trees have little to no negative allelopathic effect, or have a positive one.

*Paeonia ostii* ‘Fengdan’ (Paeoniaceae)^26^ can grow to a height of up to 2 m. It is a woody plant with important medical use and ornamental value, and is widely cultivated in China, Japan, Korea, and other countries. Its oil effectively lowers blood lipids and prevents cardiovascular diseases. The plant also has anti-inflammatory, antioxidant, and anti-tumor properties^27,28^. Its flowers are usually pink or white, and bloom in early spring, attracting many tourists and photographers. In the first few years after planting, *P. ostii* ‘Fengdan’ has no or relatively few fruits, and its crown is also small. In addition, it has no leaves from October to February of the following year. Consequently, there is potential to plant additional crops on the arable land where tree peonies are grown. Intercropping vegetables in the open spaces between trees can improve land use and provide benefits in the same year. More efficient use of arable resources, such as implementing appropriate vegetable agroforestry systems, can help address China’s land scarcity problem. These systems would also develop vegetable resources and reduce the use of pesticides and chemical fertilizers in vegetable production.

Spinach (*Spinacia oleracea*), a green leafy vegetable that is widely recognized for its nutritional benefits^29^, is rich in nutrients such as iron, calcium, vitamin A, and vitamin C, which are important for maintaining good health. Tatsoi *(Braassica campesttris*. ssp. *chinensis*), a cruciferous vegetable, has dark green leaves that are both firm and tender and provide a range of nutrients. Every 100 g of fresh spinach leaves contains up to 70 mg of vitamin C, 180 mg of calcium, iron, phosphorus, magnesium and other minerals. Its rich flavor and vibrant color make it very popular with consumers^30^. Bok choy (*Brassica campestris* ssp. *chinensis* (L.) Makino), a non-heading Chinese cabbage variety, is resistant to cold temperatures and can grow slowly even at − 10 °C^31^. Its unique characteristics include nearly round leaves with an outward curl at the tip, and a succulent, tender texture with low fiber content. Enriched in essential trace elements, this vegetable is widely grown in the northern regions of China during the winter.

In this study, the seeds of tatsoi, spinach, and bok choy were treated with aqueous extracts of roots and leaves of the tree peony cultivar ‘Fengdan’ to simulate the process of leaching from rain in autumn and winter in northern China. The effects of different concentrations of the aqueous extracts on seed germination and physiological indexes of the three vegetables were analyzed. The objectives of this work were (1) to determine the effects of aqueous extracts of ‘Fengdan’ roots and leaves on the germination and seedling growth of tatsoi, spinach, and bok choy, (2) to identify suitable vegetables for intercropping with tree peonies, and (3) to improve intercropping systems to promote the efficient use of land resources in the long-term.

## Results

### Seed germination

With respect to seed germination, low concentrations of aqueous extract of *P. ostii* ‘Fengdan’ root had a promotional effect in each of the three vegetables, whereas high concentrations of root extracts had an inhibitory effect (*P* < 0.05) (Table 1). At low concentration (5 g L^−1^) of tree peony root extract, the seed germination rate of tatsoi, spinach, and bok choy increased by 2.33%, 6.67%, and 1.15%, respectively, compared to the control. However, the seed germination rate of tatsoi, spinach, and bok choy decreased by 14.76%, 18.70%, and 21.81%, respectively, compared to the control when the concentration of root extract reached 40 g L^−1^ (Table 1). The germination rate of tatsoi was higher than that of the other two vegetables, at all concentrations of tree peony root aqueous extract.

**Table 1:** Germination and seedling vigor of three vegetables treated with *P. ostii* ‘Fengdan’ leaf and root extracts.

Vegetables	Concentration (g L^−1^)	Germination potential (%) (GP)	Seedling vigor index(SVI)	Germination index (GI)	Germination rate (GR) (%)
Tatsoi	0(CK)	75.3 ± 1.23b	49.92 ± 1.28c	70.67 ± 2.39a	91.00 ± 0.58a
	L_5_	74.7 ± 1.25b	48.07 ± 1.44c	69.57 ± 1.67a	91.33 ± 1.20a
	L_10_	64.0 ± 0.95d	39.31 ± 0.54d	55.34 ± 0.86c	84.10 ± 0.58b
	L_20_	46.0 ± 1.50ef	36.65 ± 0.63de	46.03 ± 0.58d	79.05 ± 1.31c
	L_40_	41.3 ± 0.88f	29.12 ± 0.72f	40.11 ± 0.67e	72.33 ± 1.76d
	R_5_	80.0 ± 2.05a	61.05 ± 1.67a	71.41 ± 1.76a	93.33 ± 1.30a
	R_10_	70.0 ± 1.35c	54.84 ± 0.79b	68.90 ± 2.15a	92.67 ± 0.88a
	R_20_	62.0 ± 1.07d	45.61 ± 0.94cd	63.00 ± 0.77b	85.33 ± 0.33b
	R_40_	51.0 ± 1.63e	34.44 ± 1.29e	61.93 ± 1.65b	76.24 ± 2.03cd
Spinach	0(CK)	74.7 ± 1.08a	78.03 ± 1.37b	68.26 ± 1.53a	87.00 ± 0.58b
	L_5_	64.0 ± 2.06b	78.90 ± 2.19b	69.27 ± 2.14a	92.33 ± 0.67a
	L_10_	44.0 ± 2.12cd	53.61 ± 1.26c	43.45 ± 0.42c	79.20 ± 1.73c
	L_20_	39.3 ± 1.57d	35.41 ± 0.47d	38.76 ± 1.38d	67.15 ± 1.15d
	L_40_	30.7 ± 1.39e	25.10 ± 0.59f	32.90 ± 1.46e	60.24 ± 2.52e
	R_5_	48.7 ± 1.62c	92.38 ± 3.04a	66.43 ± 2.35a	93.67 ± 0.88a
	R_10_	40.7 ± 1.73d	78.09 ± 1.75b	55.40 ± 1.05b	86.05 ± 1.15b
	R_20_	40.0 ± 1.52d	55.56 ± 1.81c	38.97 ± 2.34d	76.34 ± 1.86c
	R_40_	38.0 ± 1.29d	30.35 ± 0.94e	34.65 ± 1.08de	68.30 ± 1.20d
Bok choy	0(CK)	85.3 ± 1.57a	87.88 ± 1.27a	70.49 ± 2.24a	89.17 ± 1.86a
	L_5_	78.67 ± 1.68b	79.42 ± 2.17b	67.26 ± 0.36ab	89.67 ± 1.20a
	L_10_	72.00 ± 1.28c	52.10 ± 2.04c	61.61 ± 1.20b	76.33 ± 0.88b
	L_20_	68.00 ± 2.13cd	33.41 ± 0.72d	59.14 ± 0.69b	66.35 ± 1.20c
	L_40_	52.00 ± 1.37d	19.16 ± 0.67e	52.51 ± 1.37c	54.00 ± 3.06d
	R_5_	81.33 ± 1.34ab	89.44 ± 2.04a	70.54 ± 2.41a	90.32 ± 0.33a
	R_10_	72.00 ± 2.17c	75.26 ± 1.39b	62.68 ± 1.53b	87.00 ± 1.15ab
	R_20_	66.67 ± 2.16cd	50.55 ± 0.83c	55.16 ± 0.67c	78.02 ± 0.58b
	R_40_	44.67 ± 2.24f	30.55 ± 0.77d	39.32 ± 0.39d	67.36 ± 1.20c

L_5_, L_10_, L_20_ and L_40_ represent: 5, 10, 20 and 40 g L^−1^ of *P. ostii* ‘Fengdan’ leaf extracts, respectively. R_5_, R_10_, R_20_ and R_40_ represent: 5, 10, 20 and 40 g L^−1^ of *P. ostii* ‘Fengdan’ root extracts, respectively. The same below.

Mean ± SD, n = 3. Different lowercase letters among treatments indicate significant differences (*p* < 0.05).

Tree peony leaf extract had a greater impact than did root extract on the seed germination rate of the three vegetables. At 20 g L^−1^ leaf extract, the germination rate of tatsoi, spinach, and bok choy was reduced by 11.95%, 19.85%, and 22.82%, respectively, compared to the control, whereas at 20 g L^−1^ root extract, the germination rate was reduced by only 5.67%, 10.66%, and 11.15%, respectively, compared to the control. The germination index of tatsoi peaked at 5 g L^−1^ aqueous leaf extract and decreased with increasing concentration compared to the control. Tree peony root and leaf extracts had the strongest allelopathic effect on the germination rate of bok choy, followed by spinach and tatsoi. Overall, the germination rate of the three vegetables was in the following order: tatsoi, spinach, and bok choy.

Regardless of the leaf or root aqueous extract concentration (10–40 g L^−1^), the GR, GI, GP, and SVI were lowest in bok choy compared to the control**.** The GI and GP of spinach decreased with increasing concentration of tree peony leaf or root extract as compared to the control (Table 1).

### Seedling growth

**Fig. 1:**
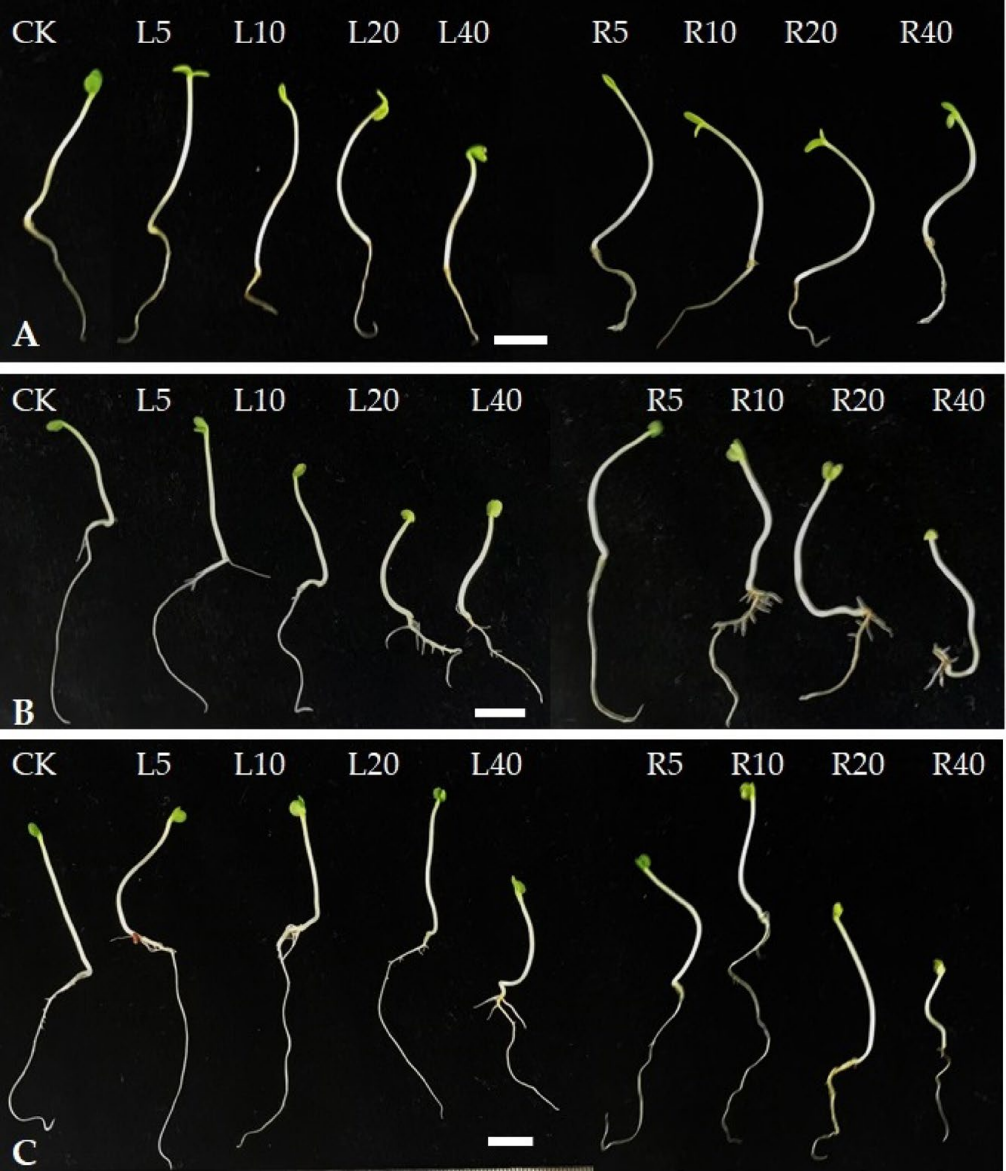
Effects of *P. ostii* ‘Fengdan’ leaf and root extracts on hypocotyl length and root length of three vegetables. (**A**) tatsoi, (**B**) spinach, (**C**) bok choy. Bar = 1.0 cm.

Leaf extract of the tree peony cultivar ‘Fengdan’ showed stronger inhibitory effects than did root extracts on the root length (RL) and hypocotyl length (HL) of tatsoi, spinach, and bok choy. Different concentrations of ‘Fengdan’ leaf extracts all had an obvious inhibitory effect on the shoot growth of the three vegetables. The higher the concentration of ‘Fengdan’ leaf extract, the stronger the inhibitory effect on the RL and HL of the vegetable seedlings. Higher concentrations of leaf extracts resulted in stronger inhibition of hypocotyl growth (Fig. 1). At 40 g L^−1^ of leaf extract, the hypocotyl length of tatsoi, spinach, and bok choy decreased by 3.10, 21.05, and 23.01 mm, respectively, compared to the corresponding measure in the control group, and the root length decreased by 13.24, 20.48, and 35.58 mm, respectively.

**Fig. 2:**
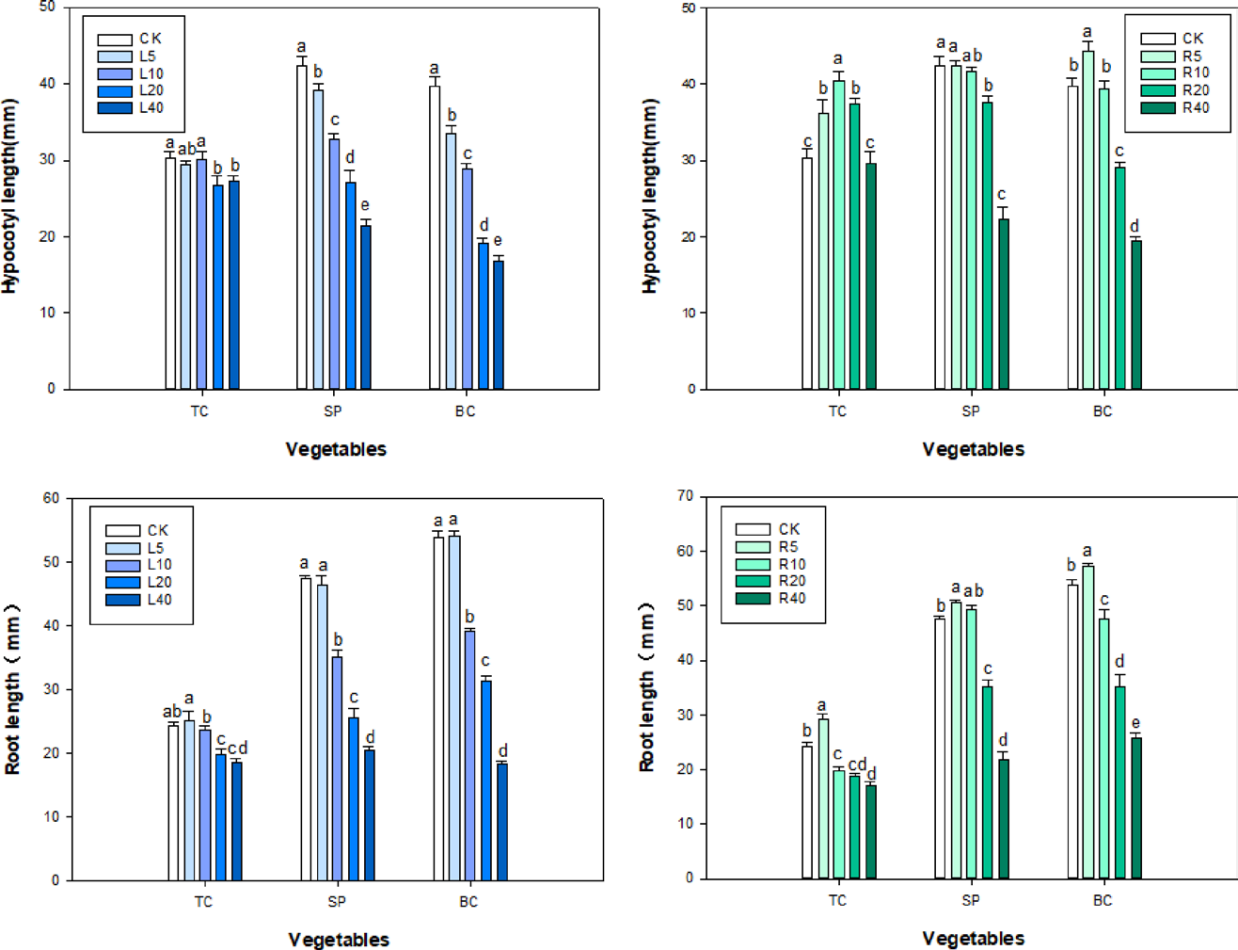
Effects of *P. ostii* ‘Fengdan’ leaf and root extracts on hypocotyl length and root length of three vegetables. (TC) tatsoi, (SP) spinach, (BC) bok choy. the same below. Different lowercase letters among treatments indicate significant differences (*p* < 0.05). The same below.

‘Fengdan’ root extract promoted the RL of tatsoi, spinach, and bok choy at 5 g L^−1^ and inhibited RL at 20–40 g L^−1^. At 5 g L^−1^ ‘Fengdan’ root extract, the RL of the three vegetables was noticeably different from that in the control seedling. On the other hand, RL of each of the three vegetables was inhibited with increasing extract concentrations of the ‘Fengdan’ leaf extracts. At 40 g L^−1^, the significant inhibitory effects were observed in the HL of the three vegetables compared to that of the control, with spinach showing the greatest reduction (54.02%) in RL compared to the control, followed by bok choy (51.98%), and tatsoi (38.19%). The changes in HL of the three vegetables treated with ‘Fengdan’ root extract showed the same trend; the root extract was found to be bio-stimulatory at low concentrations (5–10 g L^−1^), with the exception of spinach, and almost significant inhibitory impacts were recorded at 20–40 g L^−1^ of ‘Fengdan’ root extract (Figs. 1 and 2).

### FW and DW

**Fig. 3:**
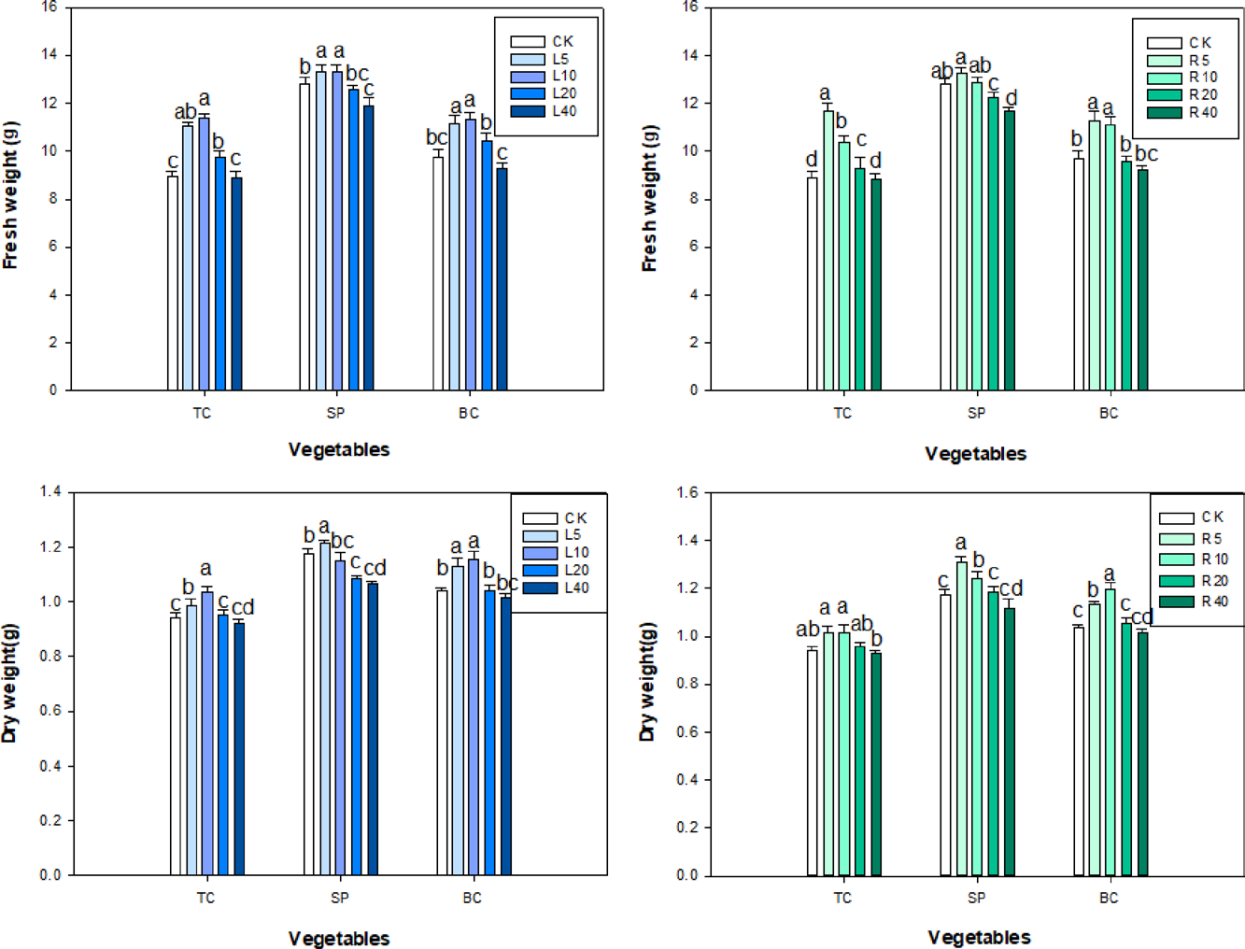
Effects of *P. ostii* ‘Fengdan’ leaf and root extracts on FW and DW in three vegetables seedlings.

The seedling FW and DW of the three vegetables varied depending on the concentration of ‘Fengdan’ leaf or root extracts. The changes in FW and DW of the three vegetables treated with ‘Fengdan’ leaf or root aqueous extracts (from 5 to 40 g L^−1^) were consistent: there was an initial increase in FW and DW followed by a decrease as the concentration of root or leaf extract increased (Fig. 3). The seedling FW values of tatsoi, spinach, and bok choy treated with 40 g L^−1^ ‘Fengdan’ leaf extract decreased by 0.09, 0.21, and 0.67 g respectively; DW decreased by 0.02, 0.10, and 0.03 g.

The FW and DW of tatsoi, spinach, and bok choy seedlings treated with 5 g L^−1^ leaf or root extract were significantly higher than the corresponding measures in the control and showed a decreasing trend with increasing concentration (Fig. 3). The FW of tatsoi seedling treated with 5–40 g L^−1^ leaf or root water extract was apparently higher than that of the control. The DW of each of the three vegetables treated with ‘Fengdan’ leaf extract was lower than DW of plants treated with the root extract. The effects of ‘Fengdan’ leaf or root extract on DW and FW of the three vegetables are in the following order: spinach, bok choy, and tatsoi.

### Pn, Tr, Ci, Gs and chlorophyll content

**Fig. 4:**
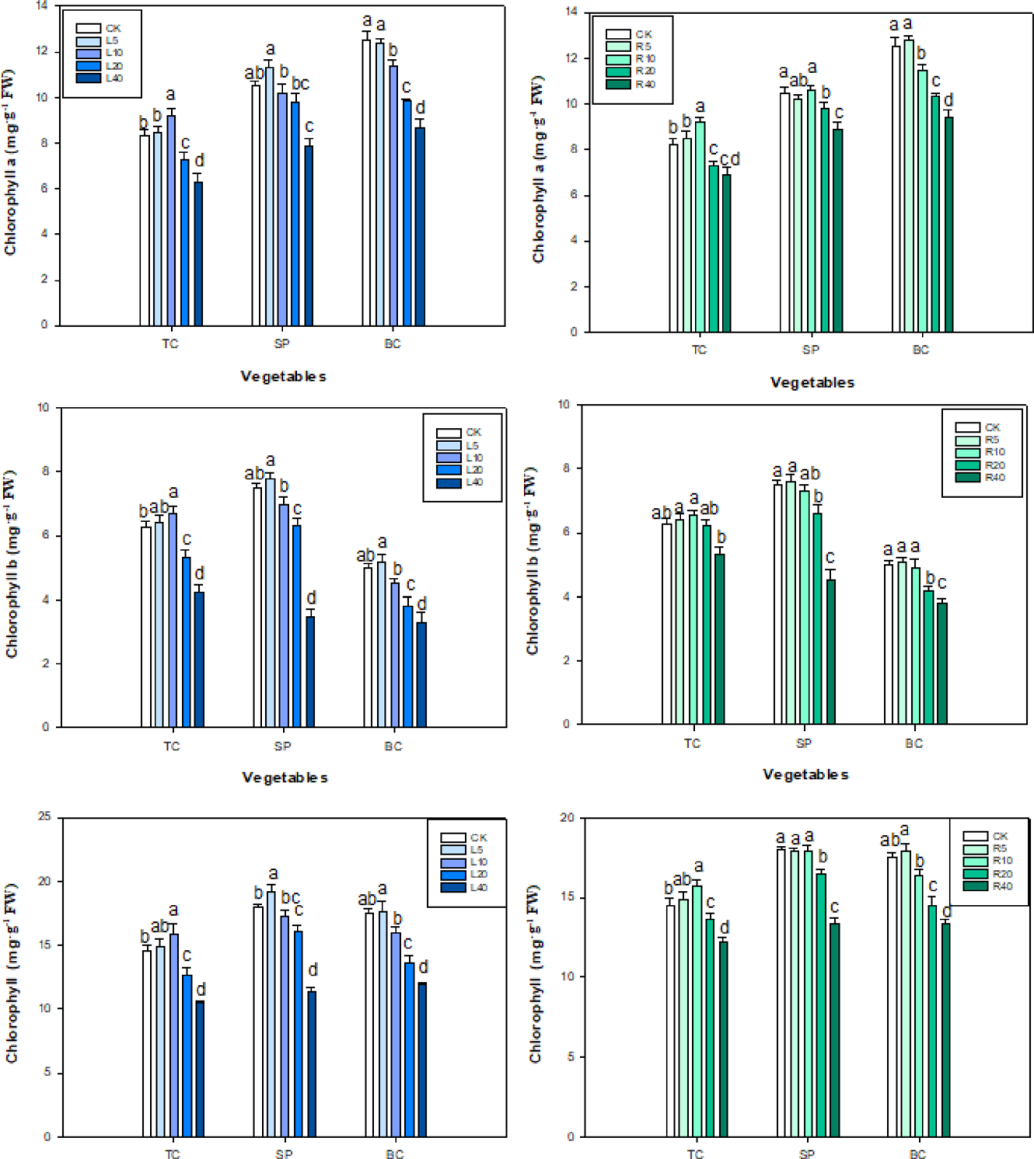
Effect of *P. ostii* ‘Fengdan’ leaf and root extracts on chlorophyll a and b in three vegetables.

In the ‘Fengdan’ aqueous leaf extract treatment groups, chlorophyll a and b in tatsoi and spinach peaked at 10 and 5 g L^−1^ leaf extract, respectively, and then declined slowly with the increase in leaf extract concentration. Chlorophyll a in spinach peaked at 5 g L^−1^ aqueous extract of ‘Fengdan’ leaves and then decreased significantly (*P* < 0.05), whereas chlorophyll b in spinach decreased gradually as the concentration of leaf aqueous extract rose (Fig. 4).

4

In the aqueous root extract treatments, chlorophyll a and b in tatsoi and bok choy peaked at 10 and 5 g L^−1^, respectively and then decreased slowly with increasing concentration of the root extract (Fig. 4). Chlorophyll a in spinach showed a tendency to first decrease, increase, and then slowly decline with the increase in the root extract; chlorophyll b in spinach peaked at 5 g L^−1^ of root extract and then decreased significantly (*P* < 0.05).

**Fig. 5:**
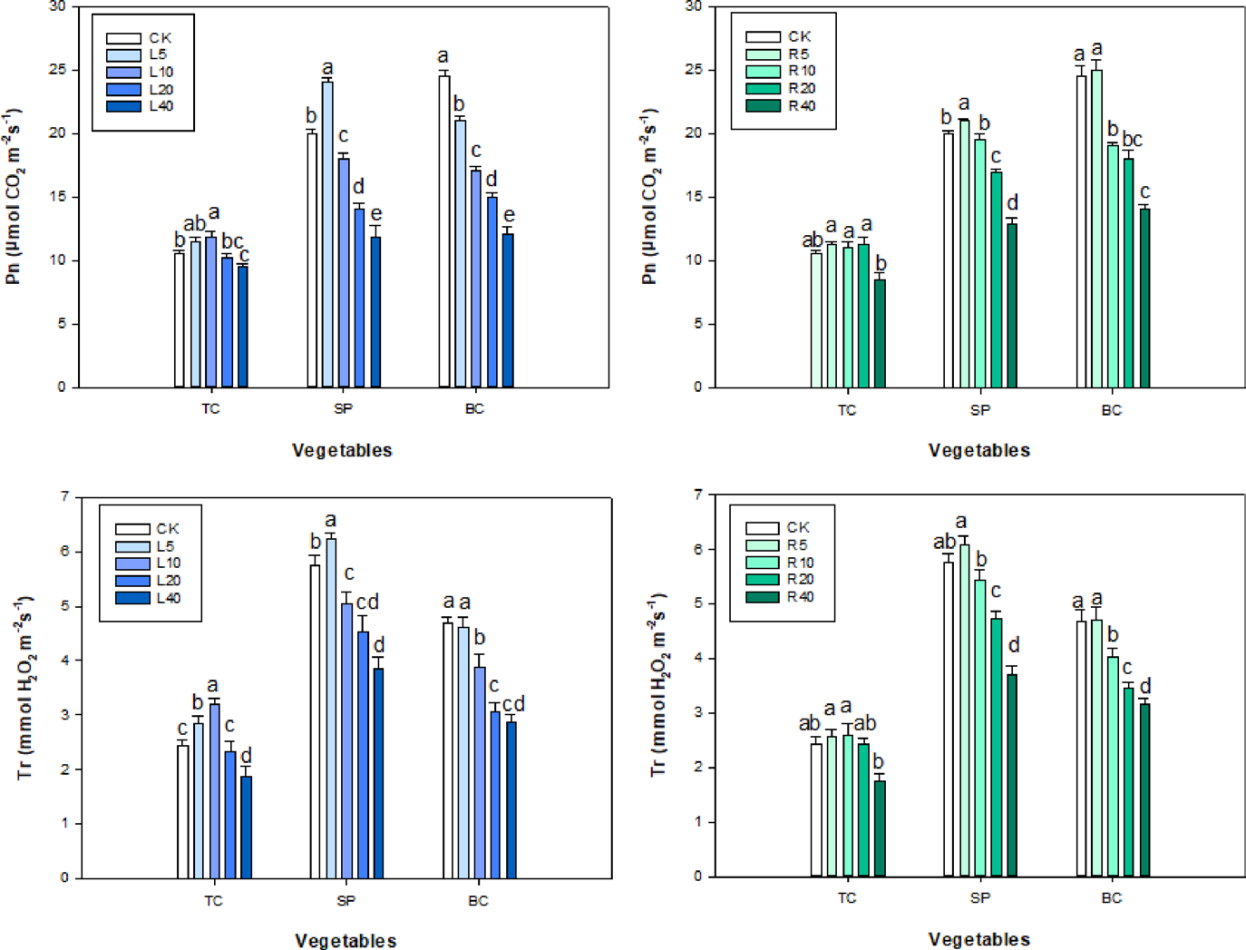
Effect of *P. ostii* ‘Fengdan’ leaf and root extracts on Pn and Tr in three vegetables.

**Fig. 6:**
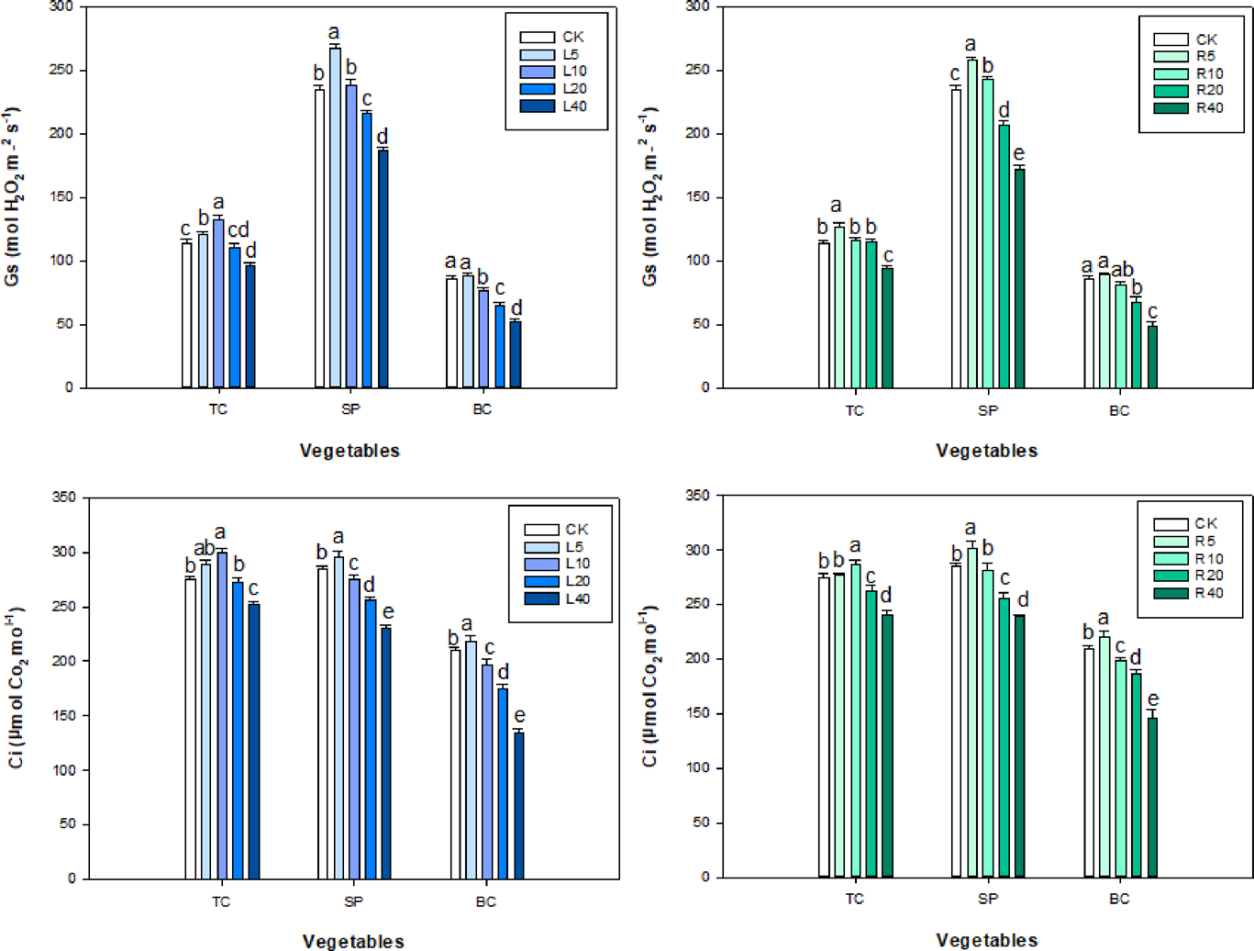
Effect of *P. ostii* ‘Fengdan’ leaf and root extracts on Gs and Ci in three vegetables.

In the aqueous leaf extract treatments, the measured values of Pn, Tr, Ci, and Gs in tatsoi and spinach peaked at 10 and 5 g L^−1^, respectively and then gradually decreased with the increase in aqueous leaf extract concentration. At 5–10 g L^−1^ the tree peony leaf extract significantly promoted Pn, Tr, Gs, and Ci values in tatsoi (increased by 0.95, 0.43, 6 and 14, respectively) (Figs. 5 and 6) (*P* < 0.05). Pn, Tr, Gs, and Ci values in bok choy were lower than the corresponding values in the control group, with the exception of the 5 g L^−1^ leaf extract treatment group. Pn, Tr, Gs, and Ci of treated bok choy were lower than the control value (Figs. 5 and 6).

In the ‘Fengdan’ root aqueous extract treatment, Pn, Tr, and Ci values in tatsoi and spinach first rose and then fell with increasing concentration of root extract. Under 20 g L^−1^ of ‘Fengdan’ root extract, Pn, Tr, Gs, and Ci in tatsoi increased only by 0.49, 0.17, 2, and 13, respectively, as compared to the control. Under 5 g L^−1^ of leaf aqueous extract, Pn, Tr, Ci, and Gs of spinach peaked, and then these four indices declined with the rise in root aqueous extract concentration. In the case of spinach seeds and plants under ‘Fengdan’ root aqueous extract treatments, Pn, Tr, Gs, and Ci values were lower than those of the control, with the exception of the 5 g L^−1^ treatment (Figs. 5 and 6).

### Root activity

**Fig. 7:**
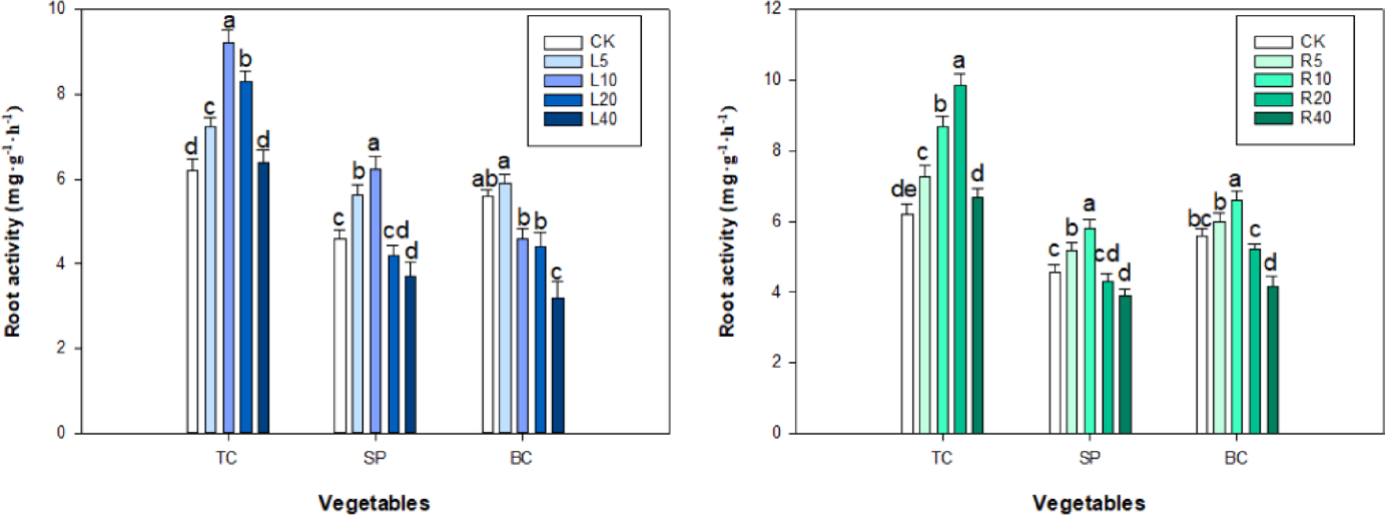
Effect of *P. ostii* ‘Fengdan’ leaf and root extracts on root activities in three vegetables.

In tatsoi seedlings, root activity responded favorably to increasing concentration of either root or leaf extract and then declined at 20 g L^−1^ (Fig. 7). Low concentration (5–10 g L^−1^) of either leaf or root extract of ‘Fengdan’ also promoted the root activity in spinach and bok choy, whereas high concentration (20–40 g L^−1^) of the ‘Fengdan’ extracts had an inhibitory effect (Fig. 7).

### Antioxidant enzyme and MDA

**Fig. 8:**
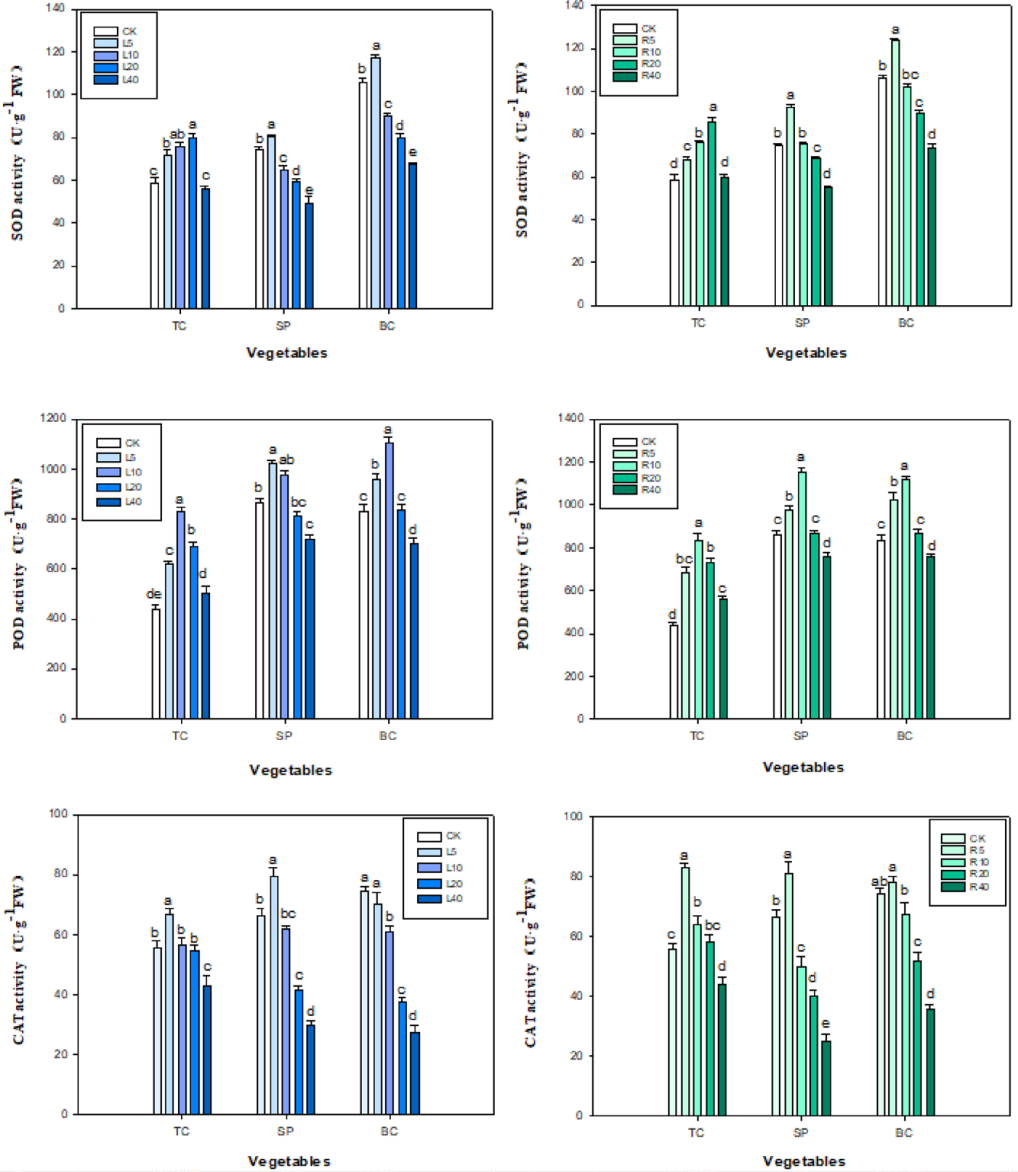
Effect of *P. ostii* ‘Fengdan’ leaf and root extracts on SOD, POD and CAT activities of three vegetables.

Both the leaf and root extract of ‘Fengdan’ were found to have a significant effect (*P* < 0.05) on the activity of SOD, POD, and CAT in tatsoi, spinach, and bok choy seedlings (Fig. 8). In the case of the leaf extract treatment, the SOD activity of spinach and bok choy seedlings showed an initial increase followed by a rapid decrease with increasing extract concentration, and decreased to 49.23 and 67.35 U g^−1^ FW, respectively, at 40 g L^−1^ of extract. The trend in the root extract treatment groups was similar: the SOD activity of spinach and bok choy seedlings gradually decreased with increasing concentration of root extract, from 5 to 40 g L^−1^, and the SOD activity at 40 g L^−1^ of root extract was 55.20 and 73.75 U g^−1^ FW, which was 73.94% and 69.20% of the control values. In tatsoi seedlings, the SOD activities increased quickly with increasing concentration of leaf and root extracts and reached a maximum at 20 g L^−1^ of concentration before decreasing to 56.00 U g^−1^ FW at 40 g L^−1^ of leaf extract and 60.05 U g^−1^ FW at 40 g L^−1^ of root extract (Fig. 8).

In the root extract treatment, CAT activity in tatsoi, spinach, and bok choy seedlings increased rapidly with increasing concentration, peaking at 5 g L^−1^ of concentration. Thereafter, CAT activity decreased rapidly with increasing concentration, reaching 83.10, 81.00, and 78.25 U g^−1^, respectively, at 40 g L^−1^ of root extract (Fig. 8). In the case of the leaf extract treatment, with increasing concentration, CAT activities in tatsoi and spinach seedlings—but not in bok choy—showed a rapid increase with increasing concentration of the extract, followed by a rapid decrease; peak values of 66.75 and 79.48 U g^−1^, respectively, were obtained at the lowest extract concentration, 5 g L^−1^, followed by rapid decrease in CAT activity, the lowest levels being 42.85 and 29.65 U g^−1^ (at 40 g L^−1^ of extract), which were 77.21% and 44.65%, respectively, of the control values.

As for the leaf extract treatment groups, POD activity in spinach seedlings gradually increased and then decreased; at 5 g L^−1^ a peak in activity occurred, 1020.5 U g^−1^ min^−1^, which was 118.32% of the control. In the case of tatsoi and bok choy, the trend in POD activity was similar; at 10 g L^−1^ of the tree peony leaf extract, the maximum POD activity values were 827.50 and 1105.20 U g^−1^ min^−1^, respectively, which were 189.48% and 132.99% of the control values. As for the root extract treatment, POD activity in tatsoi, spinach, and bok choy seedlings exhibited a trend of first rising and then declining; as extract concentration reached 10 g L^−1^, the peaked POD activity was 834.25, 1153.15, and 1120.38 g^−1^ min^−1^, respectively, which were 191.05%, 133.70%, and 334.82% of that of the control, indicating the high level of enzymatic activity in the case of POD (Fig. 8).

**Fig. 9:**
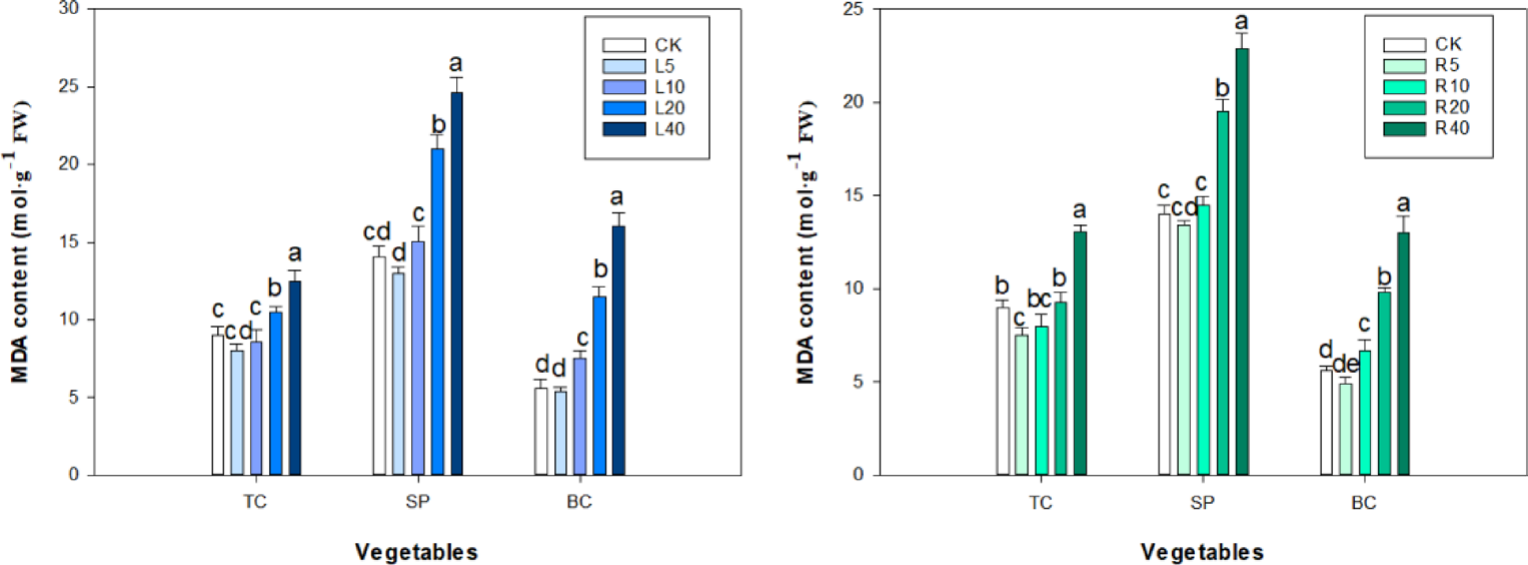
Effect of *P. ostii* ‘Fengdan’ leaf and root extracts on MDA content in three vegetables.

The basal level of MDA content in spinach seedlings was found to be significantly higher than that of tatsoi and bok choy. Different concentrations of *P. ostii* ‘Fengdan’ leaf or root extracts have a significant effect on MDA levels in tatsoi, spinach, and bok choy seedlings (Fig. 9). MDA content in three vegetable seedlings gradually decreased and then rapidly increased with increasing root and leaf extracts. At 5 g L^−1^ leaf extract, the MDA content of tatsoi, spinach, and bok choy seedlings was 88.89%, 92.72%, and 96.43% of that in control, respectively, whereas at 40 g L^−1^ leaf extract, the MDA level in tatsoi, spinach, and bok choy seedlings was 0.39, 0.75, and 1.86 times higher than that in control, respectively. Compared to the control, the MDA content of three vegetables at leaf extracts increased significantly by 3.50, 10.58, and 5.90 mol g^−1^ FW, respectively, at 40 g L^−1^ leaf extract treatment (*P* < 0.05). Among the test vegetables, bok choy seedling showed more sensitivity to the allelochemicals of leaf extracts than did the other vegetables; the significant increase in the MDA level in bok choy was observed at 40 g L^−1^ of leaf extract. The MDA content of tatsoi, spinach, and bok choy seedlings decreased at 5 g L^−1^ root extract compared to control, whereas an apparently further increase in MDA content was recorded at 10–40 g L^−1^ of root extract. MDA content in all tested vegetable seedlings was significantly increased by root extracts at higher concentrations (40 g L^−1^) (*P* < 0.05).

### Synthetic allelopathic effect

**Fig. 10:**
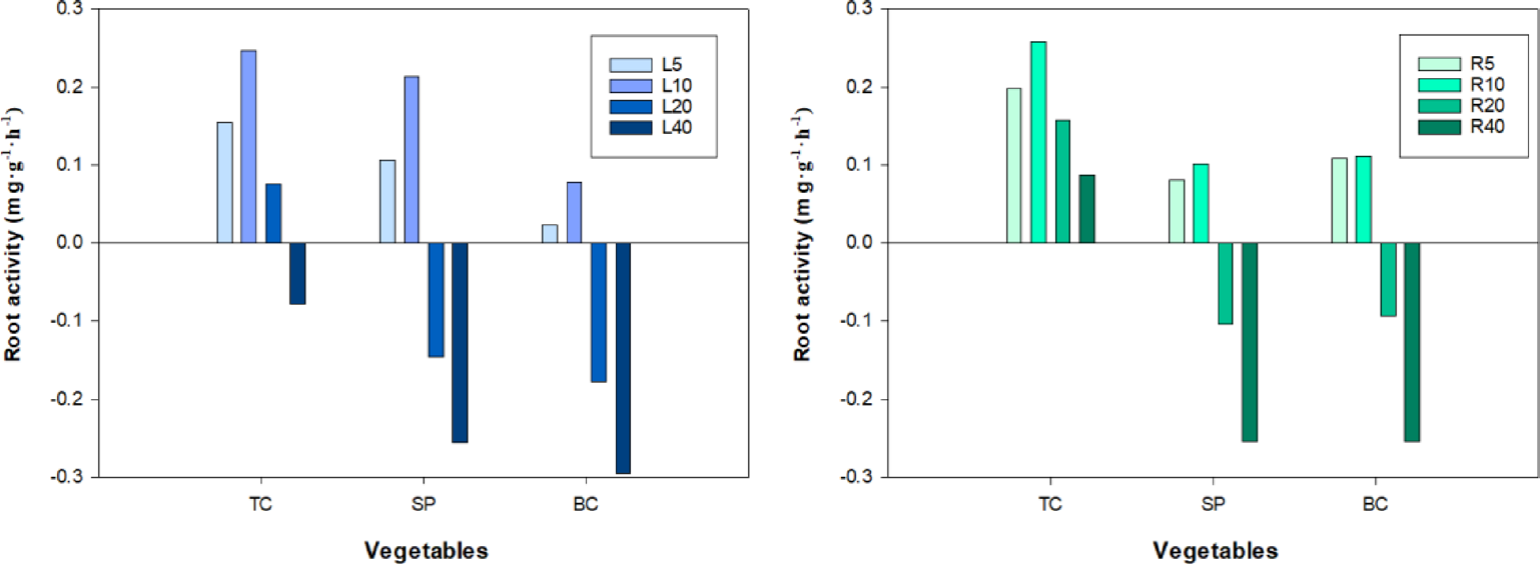
Synthetic inhibiting effect of *P. ostii* ‘Fengdan’ leaf and root extracts on three vegetables.

**Fig. 11:**
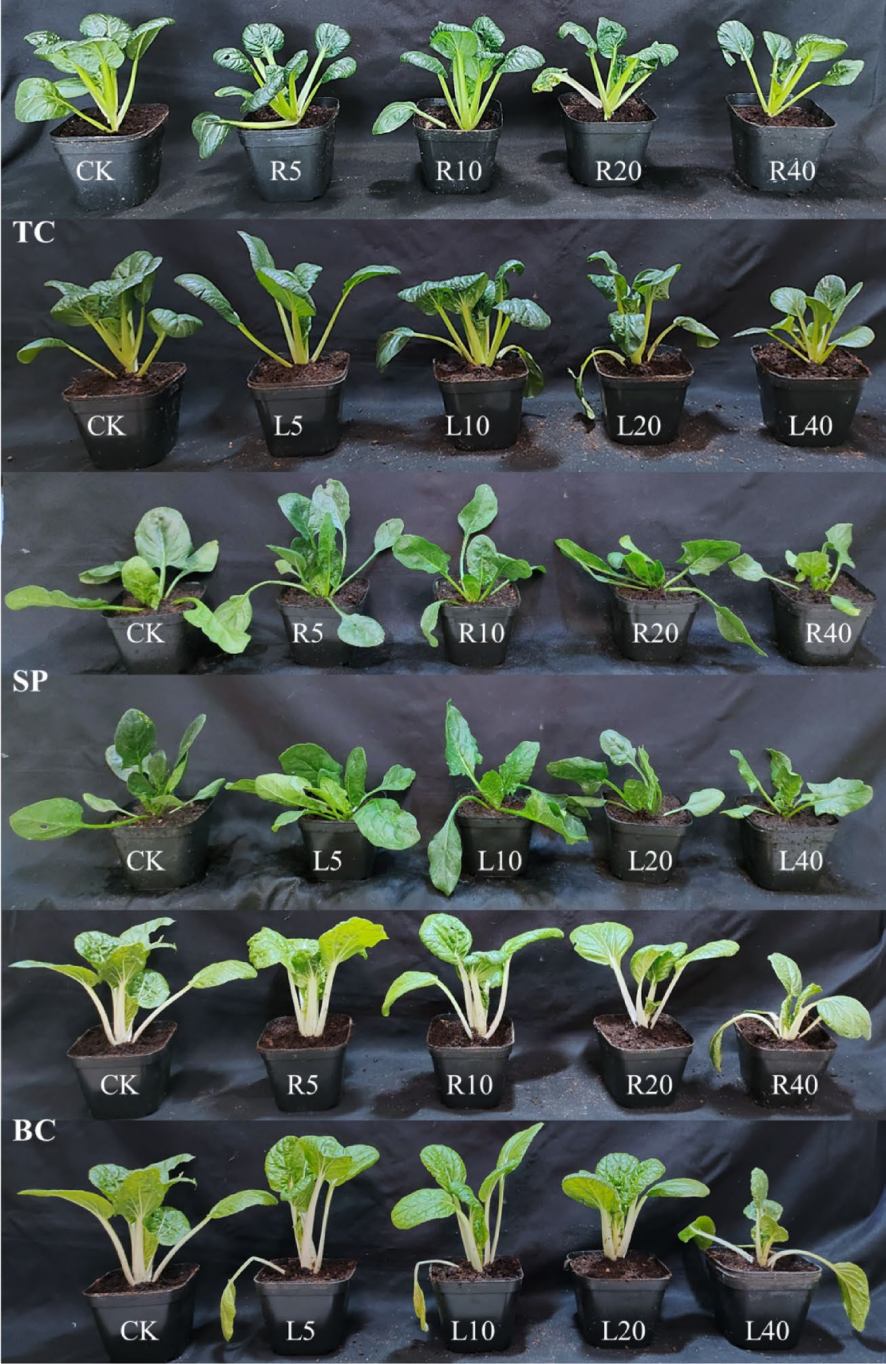
Effect of *P. ostii* ‘Fengdan’ leaf and root extracts on three vegetable seedlings.

Thirteen indicators, including GR, GI, SVI, GP, enzyme activities, MDA content, root activities, photosynthetic parameters, and chlorophyll content, were used to evaluate the synthetic allelopathy effect and to speculate the total allelopathic intensity of different concentrations of *P. ostii* ‘Fengdan’ leaf and root extracts on three vegetables (Fig. 10). 5–10 g L^−1^ of *P. ostii* ‘Fengdan’ leaf or root extracts promoted the growth of tatsoi, and the maximum synthetic allelopathic index was 0.246 at 10 g L^−1^. The synthetic allelopathic index for spinach and bok choy were negative at 20–40 g L^−1^, indicating inhibitory effects on spinach and bok choy. The order of the allelopathic effect of the root or leaf extract on the three vegetables was bok choy, spinach, and tatsoi (Fig. 11).

## Discussion

VAFS is a system in which vegetables and trees are grown together, with potential for greater economic benefits^32,33^. Different trees and vegetables differ in their ability to complement or compete in VAFS^34^. Allelopathy is an important factor affecting seed germination^35–38^. As early as 1937, H. Molisch already pointed out that it has two different effects, inhibition and promotion^39,40^. In this study, the leaf or root extracts of tree peony were used to investigate the effect on seed germination of three vegetables. Our results indicate that leaf or root extracts from *P. ostii* ‘Fengdan’ showed allelopathy effects on the HL, RL, and seed germination of three vegetables. In particular, root extract at low concentrations (5–10 g L^−1^) clearly promoted the germination rate, HL, and RL, whereas the germination was most inhibited by the leaf extract, with statistically significant results. Aqueous leaf extract of tree peony has more allelopathic effect than does aqueous root extract. The higher the concentration of *P. ostii* ‘Fengdan’ leaf or root extracts, the more intense the suppression on the germination rate of tatsoi, spinach, and bok choy seeds, which is in line with the results reported previously^36,41^. The low concentration of plant allelochemicals usually have a promoting effect on plant growth and development^42,43^. The effect of *P. ostii* ‘Fengdan’ leaf extract on tatsoi seed germination was greater than that of the root extract. However, even at high concentrations, the germination rate of tatsoi seeds remained high. This may be because tatsoi seeds are more adaptable to tree peony’s allelopathic substances (ferulic acid, cinnamic acid, vanillin coumarin, and paeonol)^44^. Tatsoi is a suitable vegetable for intercropping with tree peonies. These findings are in line with results reported in the study by Li et al.^36^; the authors found that the mint is the suitable medicinal plant for interplanting with fig trees.

Aqueous herb extracts of three plants, including *Achnatherum splendens* and *Stellera chamaejasme*, inhibited the seed germination and RL in lettuce, and promoted the growth of lettuce shoot^45^. Numerous studies have reported that the growth of receptor plant roots is more susceptible to inhibition by allelopathic substances compared to shoot growth^35,46,47^. We observed that aqueous extracts of *P. ostii* ‘Fengdan’ leaves or roots had positive effects on the growth of tatsoi, spinach, and bok choy seedlings at lower concentrations and negative effects at higher concentrations, especially at > 20 g L^−1^. *Paeonia ostii* ‘Fengdan’ root or leaf extracts inhibited the root length of the three vegetables more than their hypocotyl length. Leaf extracts had a greater influence on the shoot and root growth of the three vegetables than root extracts did. The greatest decrease in root and hypocotyl length occurred in the three vegetables at the highest concentration of aqueous *P. ostii* ‘Fengdan’ leaf extract (40 g L^−1^). The ratio of root length to shoot length declined in tatsoi, spinach, and bok choy in response to tree peony leaf or root extracts. Our findings are consistent with a previous report that the effect of *Artemisia frigida* extract was stronger on lettuce root length than shoot length^45^.

Allelochemicals can affect all aspects of plant growth, both plant physiological and biochemical processes^48–52^. The FW and DW of seedlings are the accumulation of photosynthetically produced substances in plants, and are commonly used indicators to evaluate plant growth. The study by Ludmiła et al. (2020) on mosquito motherwort (*Veronica peregrina*) showed that the positive or negative effect of chemosensitization on growth depends on the concentration of the extract: low concentration stimulates; high concentration inhibits^53^. The low concentration (8.3–12.5 g L^−1^) of fig tree leaves extract promoted the chlorophyll content, Pn, Tr, FW, DW, Gs, and Ci in mint and dandelion; the Pn, Tr, chlorophyll content, Gs, and Ci were positively correlated with the DW of mint, dandelion, and woad^36^. The suppression of aerial parts extracts on the seedling growth of the weeds increased with increasing concentrations of extracts applied^54^. Aqueous extracts of *Artemisia frigida* had hormetic effects on lettuce seedling weight^45^. Allelochemicals can have a negative effect on seedling photosynthesis, thereby reducing biomass assimilation^36^. Allelochemicals can alter the structure of soil microbial communities^36^, which can be beneficial for the growth of neighboring plants. Our results show that the 5 g L^−1^ of tree peony ‘Fengdan’ leaf extract and 5–10 g L^−1^ of root extract significantly raised the chlorophyll a and chlorophyll b content and have a promoted effects on Pn, Tr, Gs, Ci, FW, and DW of three vegetables. This suggest that some allelopathic substances in the roots and leaves of tree peonies may have promote the formation of roots, thus enhancing the growth of three vegetables. Therefore, further research is necessary to isolate and identify the allelochemicals in tree peony that promote the growth of three vegetables. Furthermore, chlorophyll content, FW, and DW of tatsoi, spinach and bok choy seedlings increased at low concentration(≤ 10 g L^−1^) of *P. ostii* ‘Fengdan’ leaf and root extracts and was inhibited at high concentrations (≥ 20 g L^−1^). At low concentrations (≤ 10 g L^−1^). This suggests that the increase in DW of the three vegetables is due to increased photosynthetic capacity resulting from higher chlorophyll content—certain allelochemicals present at higher concentrations in *P. ostii* ‘Fengdan’ leaf extract may affect the photosynthesis of tatsoi, spinach, and bok choy seedlings, resulting in a decline in FW and DW. Overall, tatsoi is the most suitable vegetable for intercropping with tree peony.

The drastic changes of antioxidant enzymes, root activity, and MDA levels in plants during intercropping have been reported to be related to allelopathic stress^15,55,56^. Normally, intracellular ROS and antioxidant molecules show a good balance in aerobic organisms^57,58^. When plants are subjected to external biological stress, a large number of ROS and MDA are produced, which severely hindered plant seedling growth, and the plant could adjust the activity of antioxidant enzymes to scavenge these harmful substances and prevent cellular damage^59^. The MDA can directly reflect the degree of damage to plant cells^60,61^. Therefore, the study of the changes in antioxidant enzyme activities and MDA levels of the three vegetables could allow us to understand the extent of the effect of *P. ostii* ‘Fengdan’ extracts on their growth. Kupidłowska et al.^62^ and Oracz et al.^63^ found that sunflower leaf extract affected the metabolic processes in cells and reduced the germination rate of white mustard seed. Chlorophyll content affects the efficiency of light energy utilization by plants^35^.

High concentrations of donor plant extracts significantly reduced chlorophyll content, root activities, the activities of antioxidant enzymes of recipient plant seedlings, whereas low concentrations of extracts stimulated their activities^35,36^. Low concentrations of leaf or root extracts of *P. ostii* ‘Fengdan’ increased root activity and chlorophyll content as well as the activity of SOD, CAT, and POD in seedlings of all three vegetables, whereas high concentrations of leaf or root extract had an inhibitory effect on root activity and CAT and POD activity. The opposite trend was observed for MDA content, indicating that the tree peony allelochemicals at higher concentrations are toxic to the vegetable, severely affecting the root vigor and photosynthesis of the seedlings, which in turn reduced the DW values of the seedlings. These results are in line with the findings of Wang et al^45^, who reported that CAT and POD decreased with increasing concentration of four shrub leaf extracts, whereas MDA showed a tendency to decrease and then increase. However, in the present study, we found that the SOD activity decreased with increasing concentration of *P. ostii* ‘Fengdan’ root or leaf extracts in both bok choy and spinach seedlings but not in tatsoi. Our findings are consistent with the results from Wang et al., who stated that the activity of SOD was significantly decreased by *Phytolacca latbenia* root extract in *Brassica napus* germinating seeds^17^. Deng et al.^64^ found that light-leafed alfalfa extracts dramatically lower SOD activity in alfalfa seedlings. Wang et al.^65^ also found that SOD activity in *Amygdalus thrpedunculata* seedlings gradually decreased with increasing concentration of leaf extracts of four woody plants, including *Pinus sylvestris* and *Populus simonii*. It is likely that the decrease in SOD activity at higher concentration of *P. ostii* ‘Fengdan’ leaf or root extract is related to the allelochemical produced from the tree peony. The leaf and root extracts of *P. ostii* ‘Fengdan’ exhibited varying levels of allelopathic activity with respect to seed germination, seedling growth, enzyme activity, malondialdehyde (MDA) content, and photosynthetic parameters in three vegetable crops. This variation may be attributed to the presence of allelochemicals such as ferulic acid, cinnamic acid, vanillin, coumarin, and paeonol in the leaf and root extracts^44^. Furthermore, the efficacy of organic or soil amendments in mitigating weed interference in the soybean cropping system is well documented^66^. These amendments have been shown to significantly influence the bioactivity of allelochemicals through sorption and degradation mechanisms within the soil environment^67^. Additional research is needed to substantiate the potential of organic amendments to mitigate the impact of *P. ostii* ‘Fengdan’ chemosensory substances on the growth of the three vegetables.

## Conclusions

Tatsoi, spinach, and bok choy varied in terms of sensitivity to the leaf and root allelochemicals of *P. ostii* ‘Fengdan’. The root or leaf aqueous extracts at low concentrations (5 g L^−1^) significant promoted seed germination as well as seedling growth, as evidenced by increases in HL, RL, FW, DW, chlorophyll content, Pn, Gs, Ci, Tr, and root activity in the three vegetables. However, the tree peony root and leaf extracts at higher concentrations (20 and 40 g L^−1^) significantly inhibited the seed germination and all of the plant growth parameters in all three vegetables. As well, enzymatic activity (SOD, POD and CAT) in seedlings of all three vegetables increased and then decreased with increasing concentration of leaf or root extracts, whereas the level of MDA decreased and significantly increased. We note that the trend in MDA level is counter to the changes in root activity and chlorophyll. spinach and bok choy were more sensitive to the tree peony allelochemicals than was tatsoi. The seedling growth in tatsoi was significantly higher than that in spinach and bok choy under treatment with the tree peony leaf or root extracts. The allelopathic effect of ‘Fengdan’ leaf extracts were greater than that of the root extracts. The order of the allelopathic effect of tree peony on the three vegetables was bok choy, spinach, and tatsoi. Based on this thorough analysis we conclude that tatsoi is the most suitable crop for intercropping with tree peony, but long-term field trials are needed for further validation. The results of this study contribute to further development of vegetable agroforestry systems, in particular the intercropping of vegetables with tree peony.

## Materials and methods

### Plant materials

The seeds of the recipient plants—tatsoi (‘Beishu’), spinach (‘Jinglu No.2’), and bok choy (‘Liangfeng’)—were brought from the Zhengzhou seed wholesale market, Zhengzhou City, Henan Province, China. Leaves and roots of ‘Fengdan’ were collected from Henan tree peony resource nursery, Xinxiang City, Henan Province, China (35.464928 N, 113.769141 E).

### Preparation of aqueous extracts solutions

The pest-free leaves and roots of *P. ostii* ‘Fengdan’ were rinsed three times with super-purified water, and dried in a blower drying oven at 30 °C for 48 h. The leaves and roots were then ground to 0.1–0.4 mm, and 160 g of either the dried root or leaf powder was soaked in 1000 mL of super-purified water and shaken at 25 °C for 48 h^17,68^. The water extract was filtered through a three-layer filter bag, after which the extract was centrifuged and the supernatant was collected; the water extract was diluted to 40 mg mL^−1^ with distilled deionized water and stored in brown bottles at 2 °C until use.

### Petri dish bioassay experiments

Tatsoi, spinach and bok choy seeds were sterilized with 2% NaClO for 3 min, after which they were washed five times with deionized distilled water. Fifty seeds of each vegetable were placed evenly on two layers of filter paper which was then placed in a 9 cm diameter Petri dish. Petri dishes were sorted by treatment: 7 mL of 5, 10, 20, or 40 g L^−1^ of either the leaf or root extract solutions. All the petri dishes were covered with lids and placed in an incubation room at 25 °C, 12 h light and 70% relative humidity. Each Petri dish was considered as an experimental unit, and each treatment was replicated six times. The number of germinated seeds of each of the three vegetables was recorded daily for 10 days. All dishes were kept moist by adding the aqueous extract solution (root or leaf, depending on the experimental group) every 24 h. Representative seedlings per dish were selected for measurement of root length and hypocotyl length. The growth parameters were determined as follows:

1. Germination rate (GR) = number of germinated seeds on the tenth day/total number of seeds × 100%.
2. Germination index (GI) = ∑ (the number of germinated seeds per day (Gt) /the number of germination days (Dt)).
3. Seedling vigor index (SVI) = (hypocotyl length (cm) × seed germination rate)/100.
4. Germination potential (GP) = the number of seed germination in the third day /the total number of seeds × 100.

### Pot experiment

To verify the results of Petri dish bioassay, 90 seedlings were randomly selected from each treatment after the germination test, and two seedlings of the same vegetable were planted in a pot (10 × 10 cm), pot containing 300 g of peat soil. The pots were randomly placed in a growth room for 45 days of cultivation, with a cultivation environment of 25 °C, 12 h light, and 70% relative humidity. Every 3 days, 100 mL of the treatment liquid (different concentrations of ‘Fengdan’ leaf or root water extract) were added to the designated pots, and the control pots was irrigated with 100 mL distilled water. After 15 days, one of the two vegetable seedlings growing in each pot was removed.

After 45 days of cultivation, representative seedlings were selected for measurement of fresh weight (FW) and dry weight (DW) of whole seedlings. The FW is the average weight of at least 6 seedlings, while DW is the average weight of the seedlings dried to a constant weight at 105 °C in an electric blast drying oven. Root activity was evaluated by the method of Wang et al.^69^. The chlorophyll content of the fresh leaves of the three vegetables was measured by applying the method detailed in Hartmut et at.^70^. The LI 6400 photosynthesis apparatus (LI-COR, USA) was used to determine the net photosynthetic rate (Pn), transpiration rate (Tr), stomatal conductance (Gs) and intercellular CO_2_ concentration (Ci) of the three vegetables according to the manufacturer’s instructions.

MDA level and enzymatic activity (SOD, POD, and CAT) were measured in the leaves of the three different vegetable seedlings. The seedlings were randomly selected from each treatment group and were ground in a mortar and pestle to homogenize fresh tissue material for the analysis of antioxidant enzyme activity and MDA level^71^. The extraction buffer solution was prepared by titrating 39.0 mL 0.2 M NaH_2_PO_4_ into 61.0 mL 0.2 M Na_2_HPO_4_ and diluting to 200 mL by adding 1% polyvinyl- polypyrrolidone (PVP). The solution was adjusted to pH 7. Seedling powder (1 g) was homogenized with 10 mL of the extraction buffer solution. The homogenates were then centrifuged in a refrigerated high-speed centrifuge at 15,000 g for 30 min at 4 °C. The supernatants were collected for the assessment of enzymatic activity. The activity levels of SOD, POD, and CAT were determined by the methods of Beauchamp et al.^72^, Lagrimin^73^ and Aebi et al.^74^, respectively. MDA level was measured using the method detailed by Velikova et al*.*^34^.

The synthetical allelopathic index (SE) was employed to represent the allelopathic effects of aqueous leaf or root extracts of *P. ostii* ‘Fengdan’ on seed germination. The arithmetic mean of the allelopathy respective index (RI) for 13 parameters was used for the purpose of detection. The parameters included GR, GI, SVI, GP, protective enzyme activities, MDA level, root activities, photosynthetic parameters, and chlorophyll content. The RI was calculated using the following formulas: RI = 1 − C/T (T ≥ C) or RI = T/C–1 (T < C). T: treatment, C: control. When RI > 0, it means there is a promotion effect, whereas when RI < 0, it means there is an inhibitory effect.

### Statistical analysis

All data from the Petri dish experiment and pot experiment are expressed as the mean ± standard deviation of at least six replicates. One-way ANOVA was performed for germination data, hypocotyl length, root length, FW, DW, antioxidant enzyme activity, MDA level, Pn, Tr, Gs, Ci and Chlorophyll content. Graphs were generated using SigmaPlot 14.0.

## Data Availability

All data are presented in the article, and can be requested from the corresponding author if required.

## References

[1] Ain, Q., Mushtaq, W., Shadab, M. & Siddiqui, M. Allelopathy: An alternative tool for sustainable agriculture. *Physiol. Mol. Biol. Plants* **29**, 495–511. 10.1007/s12298-023-01305-9 (2023). 37187777 10.1007/s12298-023-01305-9 PMC10172429

[2] Kostina-Bednarz, M., Płonka, J. & Barchanska, H. Allelopathy as a source of bioherbicides: Challenges and prospects for sustainable agriculture. *Rev. Environ. Sci. Bio/Technol.* **22**, 471–504. 10.1007/s11157-023-09656-1 (2023).

[3] Hoang, L. T., Roshetko, J. M., Huu, T. P., Pagella, T. & Mai, P. N. Agroforestry-the most resilient farming system for the hilly Northwest of Vietnam. *Int. J. Agric. Syst.* **5**, 1–23. 10.20956/ijas.v5i1.1166 (2017).

[4] Gautam, D. K., Bajracharya, R. M. & Sitaula, B. K. Effects of biochar and farm yard manure on soil properties and crop growth in an agroforestry system in the Himalaya. *Sustain. Agric. Res.* **6**, 74. 10.5539/sar.v6n4p74 (2017).

[5] Mercado Jr, A. R., Arcinal, G. A., Duque, C., Palada, M. C. & Reyes, M. R. Vegetable-agroforestry (VAF) system: understanding vegetable-tree interaction is a key to successful vegetable farming enterprise. *SANREM CRSP Annual Report Year 3* (Claveria, Misamis Oriental, The Philippines: World Agroforestry Center) (2008).

[6] Bijalwan, A. et al. Trends and insights of agroforestry practices in Madhya Pradesh, India. *Curr. Sci.* **117**, 597–605. 10.18520/cs/v117/i4/597-605 (2019).

[7] Lal, N. & Biswas, A. Allelopathic interaction and eco-physiological mechanisms in agri-horticultural systems: A review. *Erwerbs-obstbau* **65**, 1861–1872. 10.1007/s10341-023-00864-1 (2023).

[8] Devi, M. Allelopathy in agroforestry: A review. *J. Pharmacogn. Phytochem.* **6**, 686–688 (2017).

[9] Gonçalves, B., Morais, M. C., Pereira, S., Mosquera-Losada, M. R. & Santos, M. Tree–crop ecological and physiological interactions within climate change contexts: A mini-review. *Front. Ecol. Evol.* **9**, 661978. 10.3389/fevo.2021.661978 (2021).

[10] Kugedera, A. & Kokerai, L. Agroforestry species have negative allelopathic effect on food and fodder crops. *Int. J. Agric. Agribus.* **2**, 78–83 (2019).

[11] Krishna, A., Manjunath, G., Ramesh Rathod, R. R. & Siddappa Kannur, S. K. Allelopathic effect of four agroforestry tree species leaf leachates on seed germination of certain vegetable crops. *J. Agric. Sci.* **16**, 430–433 (2003).

[12] Lawrence, J. G., Colwell, A. & Sexton, O. J. The ecological impact of allelopathy in *Ailanthus**Altissima* (Simaroubaceae). *Am. J. Bot.* **78**, 948–958. 10.1002/j.1537-2197.1991.tb14498.x (1991).

[13] Bauer, J. T., Shannon, S. M., Stoops, R. E. & Reynolds, H. L. Context dependency of the allelopathic effects of *Lonicera**maackii* on seed germination. *Plant Ecol.* **213**, 1907–1916. 10.1007/s11258-012-0036-2 (2012).

[14] Chou, C. H. & Yang, C. M. Allelopathic research of subtropical vegetation in Taiwan II. Comparative exclusion of understory by Phyllostachys edulis and *Cryptomeria**japonica*. *J. Chem. Ecol.* **8**, 1489–1507. 10.1007/bf00989105 (1982). 24414892 10.1007/BF00989105

[15] Wang, S., Zhao, Y., Guo, J. & Liu, Y. Antioxidative response in leaves and allelochemical changes in root exudates of *Ricinus**communis* under Cu, Zn, and Cd stress. *Environ. Sci. Pollut. Res.* **25**, 32747–32755. 10.1007/s11356-018-3283-5 (2018). 10.1007/s11356-018-3283-5 30244445

[16] Singh, N. Alleviation of Allelopathic Stress of benzoic acid by indole acetic acid in *Solanum**lycopersicum*. *Sci. Hortic.* **192**, 211–217. 10.1016/j.scienta.2015.06.013 (2015).

[17] Ullah, N., Haq, I. U., Safdar, N. & Mirza, B. Physiological and biochemical mechanisms of allelopathy mediated by the allelochemical extracts of *Phytolacca**latbenia* (Moq.) H. Walter. *Toxicol. Ind. Health* **31**, 931–937. 10.1177/0748233713483205 (2015). 23572390 10.1177/0748233713483205

[18] Cakmak, I. & Horst, W. J. Effect of aluminium on lipid peroxidation, superoxide dismutase, catalase, and peroxidase activities in root tips of soybean (Glycine max). *Physiol. Plant.* **83**, 463–468. 10.1111/j.1399-3054.1991.tb00121.x (1991).

[19] Dumanović, J., Nepovimova, E., Natić, M., Kuča, K. & Jaćević, V. The significance of reactive oxygen species and antioxidant defense system in plants: A concise overview. *Front. Plant Sci.* **11**, 552969. 10.3389/fpls.2020.552969 (2021). 33488637 10.3389/fpls.2020.552969 PMC7815643

[20] Lin, C. C. & Kao, C. H. Effect of NaCl stress on H_2_O_2_ metabolism in rice leaves. *Plant Growth Regul.* **30**, 151–155. 10.1023/A:1006345126589 (2000).

[21] Cuypers, A., Vangronsveld, J. & Clijsters, H. Peroxidases in roots and primary leaves of *Phaseolus**vulgaris* copper and zinc phytotoxicity: A comparison. *J. Plant Physiol.* **159**, 869–876. 10.1078/0176-1617-00676 (2002).

[22] Yan, Z. et al. Phytotoxicity mechanisms of two coumarin allelochemicals from *Stellera**chamaejasme* in lettuce seedlings. *Acta Physiol. Plant.* **38**, 1–10. 10.1007/s11738-016-2270-z (2016).

[23] Sun, X. et al. Allelopathic effects of pyrogallic acid secreted by submerged macrophytes on *Microcystis**aeruginosa*: Role of ROS generation. *Allelopath. J.* **33**, 121–129 (2014).

[24] Chen, F. et al. Effect of plant allelochemicals on seed germination and its ecological significance. *Chin. J. Eco-Agric.* **25**, 36–46 (2017).

[25] Cai, F. et al. Lipid peroxidation and antioxidant responses during seed germination of *Jatropha**curcas*. *Int. J. Agric. Biol.* **13**, 25–30 (2011).

[26] Wang, C. et al. Effect of spatial position on twig resource allocation of Fengdan (*Paeonia**Ostii*). *Ann. Appl. Biol.* **181**, 347–356. 10.1111/aab.12780 (2022).

[27] He, C., Zhang, K., Hou, X., Han, D. & Wang, S. Foraging behavior and pollination efficiency of *Apis**mellifera* L. on the oil tree peony ‘Feng Dan’ (*Paeonia**Ostii* T. Hong et JX Zhang). *Insects* **10**, 116. 10.3390/insects10040116 (2019). 31027183 10.3390/insects10040116 PMC6523710

[28] He, C. et al. Foraging behavior of honeybees (*Apis**mellifera* L.) and ground bumblebees (*Bombus**terrestris* L.) and its influence on seed yield and oil quality of oil tree peony cultivar ‘Fengdan’ (*Paeonia**ostii* T. Hong et JX Zhang). *J. Apicultural Sci.* **64**, 131–142. 10.2478/jas-2020-0014 (2020).

[29] Roberts, J. L. & Moreau, R. Functional properties of tacai (*Spinacia**oleracea* L.) phytochemicals and bioactives. *Food Funct.* **7**, 3337–3353. 10.1039/c6fo00051g (2016). 27353735 10.1039/c6fo00051g

[30] Song, B., Xu, H., Chen, L. Z. & Yuan, X. H. Comparison of heterosis between ogura male sterile line combination and maintainer line combination in wutai vegetable. *Jiangsu Agric. Sci.* **12**, 197–199 (2015).

[31] Honma, S. & Heeckt, O. Results of crossing *Brassica**pekinensis* (Lour.) Rupr. with *B*. *oleracea* L. Var Acephala Dc. *Euphytica* **9**, 243–246. 10.1007/BF00022229 (1960).

[32] de Oliveira, J. et al. Polycultivation and agroforestry systems impact the vegetative growth of vegetables. *Rev. Agric. Neotropical.* **10**, e7544–e7544. 10.32404/rean.v10i4.7544 (2023).

[33] Bhat, S. A. Effect of tree spacing and organic manures on growth and yield of vegetable crops under melia composita willd. based agroforestry system. Ph.D Thesis, Dr. Yashwant Singh Parmar University of Horticulture and Forestry, Nauni, Solan, H.P (2015).

[34] Velikova, V., Yordanov, I. & Edreva, A. Oxidative stress and some antioxidant systems in acid rain-treated bean plants: Protective role of exogenous polyamines. *Plant Sci.* **151**, 59–66 (2000).

[35] Ming, Y. et al. Allelopathic effects of *Castanea**henryi* aqueous extracts on the growth and physiology of *Brassica**pekinensis* and Zea mays. *Chem. Biodivers.* **17**, e2000135. 10.1002/cbdv.202000135 (2020). 32249503 10.1002/cbdv.202000135

[36] Li, C. et al. The effects of fig tree (*Ficus**carica* l.) leaf aqueous extract on seed germination and seedling growth of three medicinal plants. *Agronomy* **11**, 2564. 10.3390/agronomy11122564 (2021).

[37] Kato-Noguchi, H. & Kurniadie, D. Allelopathy and allelopathic substances of mango (*Mangifera**indica* L.). *Weed Biol. Manag.* **20**, 131–138. 10.1111/wbm.12212 (2020).

[38] Prati, D. & Bossdorf, O. Allelopathic inhibition of germination by *Alliaria**petiolata* (Brassicaceae). *Am. J. Bot.* **91**, 285–288. 10.3732/ajb.91.2.285 (2004). 21653384 10.3732/ajb.91.2.285

[39] Narwal, S., Sampietro, D., Catalán, C., Vattuone, M. & Politycka, B. *Plant bioassays*; Studium Press (India), ISBN 1-62699-220-7 (2009).

[40] Molisch, H. *The Influence of One Plant on Another: Allelopathy.*; Scientific publishers (India), ISBN 81-7233-285-8 (2001;).

[41] Możdżeń, K., Barabasz-Krasny, B., Zandi, P., Kliszcz, A. & Puła, J. Effect of aqueous extracts from *Solidago**canadensis* L. leaves on germination and early growth stages of three cultivars of *Raphanus**sativus* L. Var *Radicula**Pers*. *Plants* **9**, 1549. 10.3390/plants9111549 (2020). 33198139 10.3390/plants9111549 PMC7697618

[42] Deng, Y., Zhao, Y., Padilla-Zakour, O. & Yang, G. Polyphenols, antioxidant and antimicrobial activities of leaf and bark extracts of *Solidago**canadensis* L.. *Ind. Crops Prod.* **74**, 803–809. 10.1016/j.indcrop.2015.06.014 (2015).

[43] Judžentienė, A., Būdienė, J., Labanauskas, L., Stancelytė, D. & Nedveckytė, I. Allelopathic activity of Canadian goldenrod (*Solidago**canadensis* L.) extracts on seed germination and growth of lettuce (*Lactuca**sativa* L.) and garden pepper cress (*Lepidium**sativum* L.). *Plants* **12**, 1421. 10.3390/plants12071421 (2023). 37050047 10.3390/plants12071421 PMC10096748

[44] Tan, Y. M. et al. Detection of *Paeonia**ostii* autotoxins and their mechanism. *Acta Ecol. Sin.* **29**, 1153–1161 (2009).

[45] Wang, K. et al. Aqueous extracts of three herbs allelopathically inhibit lettuce germination but promote seedling growth at low concentrations. *Plants* **11**, 486. 10.3390/plants11040486 (2022). 35214819 10.3390/plants11040486 PMC8877897

[46] Mishra, J. Allelopathic effect of *Asphodelus**tenuifolius* on wheat, mustard, lentil and chickpea. *Pestology* **25**, 48–50 (2001).

[47] Abu-Romman, S. Differential allelopathic expression of different plant parts of *Achillea**biebersteinii*. *Acta Biol. Hung.* **67**, 159–168. 10.1556/018.67.2016.2.4 (2016). 27165527 10.1556/018.67.2016.2.4

[48] Huang, Y., Bai, Y., Wang, Y. & Kong, H. Allelopathic effects of the extracts from an invasive species *Solidago**canadensi*s L. on *Microcystis**aeruginosa*. *Lett. Appl. Microbiol.* **57**, 451–458. 10.1111/lam.12133 (2013). 23848059 10.1111/lam.12133

[49] Borowiak, K. Morphological changes in two tobacco and petunia cultivars under exposure to tropospheric ozone. *Acta Biol. Cracov. Bot.* **55**, 58–66. 10.2478/abcsb-2013-0002 (2013).

[50] Mallik, A. U., Biswas, S. R. & Collier, L. C. S. Belowground interactions between *Kalmia**angustifolia* and *Picea**mariana*: Roles of competition, root exudates and ectomycorrhizal association. *Plant Soil* **403**, 471–483. 10.1007/s11104-016-2819-z (2016).

[51] Tommasino, E. et al. Malondialdehyde content as a potential biochemical indicator of tolerant *Cenchrus**ciliaris* L. Genotypes under heat stress treatment. *Grass Forage Sci.* **67**, 456–459. 10.1111/j.1365-2494.2012.00851.x (2012).

[52] Maxwell, K. & Johnson, G. N. Chlorophyll fluorescence: A practical guide. *J. Exp. Bot.* **51**, 659–668. 10.1093/jxb/51.345.659 (2000). 10938857 10.1093/jxb/51.345.659

[53] Polechońska, L., Gleńsk, M., Klink, A., Dambiec, M. & Dajdok, Z. Allelopathic potential of invasive wetland plant *Veronica**peregrina*. *Plant Biosyst. :Int. J. Deal. Aspect. Plant Biol.* **154**, 481–487. 10.1080/11263504.2019.1635225 (2020).

[54] Kordali, S., Kabaagac, G., Sen, İ, Yilmaz, F. & Najda, A. Phytotoxic effects of three origanum species extracts and essential oil on seed germinations and seedling growths of four weed species. *Agronomy* **12**, 2581. 10.3390/agronomy12102581 (2022).

[55] Weir, T. L., Park, S. W. & Vivanco, J. M. Biochemical and physiological mechanisms mediated by allelochemicals. *Curr. Opin. Plant Biol.* **7**, 472–479. 10.1016/j.pbi.2004.05.007 (2004). 15231272 10.1016/j.pbi.2004.05.007

[56] Shen, Y. et al. Role of nano-biochar in attenuating the allelopathic effect from *Imperata**cylindrica* on rice seedlings. *Environ. Sci. Nano* **7**, 116–126. 10.1039/c9en00828d (2020).

[57] He, L. et al. Antioxidants maintain cellular redox homeostasis by elimination of reactive oxygen species. *Cell. Physiol. Biochem.* **44**, 532–553. 10.1159/000485089 (2017). 29145191 10.1159/000485089

[58] Lushchak, V. I. Free radicals, reactive oxygen species, oxidative stress and its classification. *Chem. Biol. Interact.* **224**, 164–175. 10.1016/j.cbi.2014.10.016 (2014). 25452175 10.1016/j.cbi.2014.10.016

[59] Waszczak, C., Carmody, M. & Kangasjärvi, J. Reactive oxygen species in plant signaling. *Annu. Rev. Plant Biol.* **69**, 209–236. 10.1146/annurev-arplant-042817-040322 (2018). 29489394 10.1146/annurev-arplant-042817-040322

[60] Ahmed, I. M. et al. Difference in yield and physiological features in response to drought and salinity combined stress during anthesis in tibetan wild and cultivated barleys. *PLoS ONE* **8**, e77869. 10.1371/journal.pone.0077869 (2013). 24205003 10.1371/journal.pone.0077869 PMC3812012

[61] Baniasadi, F., Saffari, V. R. & Moud, A. A. M. Physiological and growth responses of *Calendula**officinalis* L. plants to the interaction effects of polyamines and salt stress. *Sci. Hortic.* **234**, 312–317. 10.1016/j.scienta.2018.02.069 (2018).

[62] Kupidłowska, E. et al. Impact of sunflower (*Helianthus**annuus* L.) extracts upon reserve mobilization and energy metabolism in germinating mustard (*Sinapis**alba* L.) seeds. *J. Chem. Ecol.* **32**, 2569–2583. 10.1007/s10886-006-9183-z (2006). 17131190 10.1007/s10886-006-9183-z

[63] Oracz, K. et al. Induction of oxidative stress by sunflower phytotoxins in germinating mustard Seeds. *J. Chem. Ecol.* **33**, 251–264. 10.1007/s10886-006-9222-9 (2007). 17216362 10.1007/s10886-006-9222-9

[64] Deng, L. et al. Alfalfa leaf-derived porous heteroatom-doped carbon materials as efficient cathodic catalysts in microbial fuel cells. *ACS Sustain. Chem. Eng.* **5**, 9766–9773. 10.1021/acssuschemeng.7b01585 (2017).

[65] Wang, X. et al. The effects of leaf extracts of four tree species on *Amygdalus**pedunculata* seedlings growth. *Front. Plant Sci.* **11**, 587579. 10.3389/fpls.2020.587579 (2021). 33584742 10.3389/fpls.2020.587579 PMC7873849

[66] Saberali, S. F. & Mohammadi, K. Organic amendments application downweight the negative effects of weed competition on the soybean yield. *Ecol. Eng.* **82**, 451–458. 10.1016/j.ecoleng.2015.05.038 (2015).

[67] Galán-Pérez, J. A., Gámiz, B. & Celis, R. Soil modification with organic amendments and organo-clays: Effects on sorption, degradation, and bioactivity of the allelochemical scopoletin. *J. Environ. Manage.* **302**, 114102. 10.1016/j.jenvman.2021.114102 (2022). 34800766 10.1016/j.jenvman.2021.114102

[68] Wang, X. et al. Allelopathic effects of aqueous leaf extracts from four shrub species on seed germination and initial growth of *Amygdalus**pedunculata* Pall. *Forests* **9**, 711. 10.3390/f9110711 (2018).

[69] Wang, C. et al. Allelopathic effects of volatile compounds from eucalyptus grandis on *Vigna**radiata*, *Raphanus**sativus* and *Lactuca**sativa*. *Allelopath. J.* **36**, 273–282 (2015).

[70] Hartmut, K. & Alan, R. Determinations of total carotenoids and chlorophylls b of leaf extracts in different solvents. *Analysis* **4**, 142–196. 10.1042/bst0110591 (1983).

[71] Nayyar, H. & Gupta, D. Differential sensitivity of C3 and C4 plants to water deficit stress: Association with oxidative stress and antioxidants. *Environ. Exp. Bot.* **58**, 106–113. 10.1016/j.envexpbot.2005.06.021 (2006).

[72] Beauchamp, C. & Fridovich, I. Superoxide dismutase: improved assays and an assay applicable to acrylamide gels. *Anal. Biochem.* **44**, 276–287. 10.1016/0003-2697(71)90370-8 (1971). 4943714 10.1016/0003-2697(71)90370-8

[73] Lagrimini, L. M. Wound-induced deposition of polyphenols in transgenic plants overexpressing peroxidase. *Plant Physiol.* **96**, 577–583. 10.1104/pp.96.2.577 (1991). 16668224 10.1104/pp.96.2.577 PMC1080809

[74] Aebi, H. Catalase in vitro. *Methods Enzymol.* **105**, 121–126 (1984). 6727660 10.1016/s0076-6879(84)05016-3

